# Structural Comparison of Diplonemid Communities around the Izu Peninsula, Japan

**DOI:** 10.1264/jsme2.ME21012

**Published:** 2021-06-11

**Authors:** Akinori Yabuki, Masaru Kawato, Yuriko Nagano, Shinji Tsuchida, Takao Yoshida, Yoshihiro Fujiwara

**Affiliations:** 1 Deep-Sea Biodiversity Research Group, Research Institute for Global Change, Japan Agency for Marine-Earth Science and Technology (JAMSTEC), 2–15 Natsushima, Yokosuka, Kanagawa 2370061, Japan

**Keywords:** community structure, cosmopolitan species, eDNA, diplonemid

## Abstract

Diplonemea (diplonemids) is one of the most abundant and species-rich protist groups in marine environments; however, their community structures among local and seasonal samples have not yet been compared. In the present study, we analyzed four diplonemid community structures around the Izu Peninsula, Japan using barcode sequences amplified from environmental DNA. These sequences and the results of statistical analyses indicated that communities at the same site were more similar to each other than those in the same season. Environmental variables were also measured, and their influence on diplonemid community structures was examined. Salinity, electrical conductivity, and temperature, and their correlated variables, appeared to influence the structures of diplonemid communities, which was consistent with previous findings; however, since the results obtained did not reach statistical significance, further studies are required. A comparison of each diplonemid community indicated that some lineages were unique to specific samples, while others were consistently detected in all samples. Members of the latter type are cosmopolitan candidates and may be better adapted to the environments of the studied area. Future studies that focus on the more adaptive members will provide a more detailed understanding of the mechanisms by which diplonemids are widely distributed in marine environments and will facilitate their utilization as indicator organisms to monitor environmental changes.

The protist community structures of various samples have been analyzed (*e.g.*, Brisbin *et al.*, 2020; Iniesto *et al.*, 2021), and differences among these samples were successfully characterized. A comparison of the bulk structure of an entire protist community is important for understanding structural differences at the large-scale level. However, major eukaryotic supergroups or higher protist taxa (*e.g.*, phylum or class) contain a wide range of members and their ecological functions are highly diverse; even within a single protist taxon, some members are photosynthetic algae and others are phagotrophic predators of bacteria. Difficulties are associated with distinguishing these functional differences based on limited molecular sequence information because members with diverse ecological functions are closely related in the phylogenetic trees of some protist groups (*e.g.*, stramenopiles and Alveolata). Furthermore, many uncultivated lineages have not yet been identified, and a clear understanding of their physiological characterization is required to comprehensively characterize the protist community (Keeling and del Campo, 2017). Although recent studies targeted broad eukaryotic lineages, a careful comparison of the community structure of a certain protist group, the members of which have similar ecological functions, may provide insights into the detection of structural differences at a finer scale.

Diplonemea (diplonemids) is a subgroup of Euglenozoa (Adl *et al.*, 2019) and currently contains three families (Diplonemidae, Hemistasiidae, and Eupelagonemidae) (Cavalier-Smith, 2016; Okamoto *et al.*, 2019). Its members have similar ecological functions, such as potential predators and scavengers of microorganisms, including zooplankton and algae (Lukeš *et al.*, 2015), whereas many environmental lineages, including deep-sea and pelagic diplonemids, have not yet been formally described (Flegontova *et al.*, 2016; Gawryluk *et al.*, 2016). Since they are counted as the 3^rd^ most diverse and 6^th^ most abundant eukaryotic group in marine environments (Lukeš *et al.*, 2015), diplonemids, including undescribed members, are common and major components of marine microbial ecosystems. The diplonemid communities of several open-ocean samples have been compared, and possible environmental variables influencing their community structures have been discussed (Flegontova *et al.*, 2020). However, this study was conducted with only one replicate of each sample, and, thus, seasonal differences in community structures using samples from the same locality have not yet been investigated. A detailed comparison of the sequences detected among samples has still not been conducted and their possible cosmopolitan and/or endemic lineages have not been discussed. These comparisons will contribute to our understanding of which lineages and species are more adaptive and widely distributed. Since certain protists have recently been the focus of research on biological indicators for environmental monitoring based on their distribution (*e.g.*, Pawlowski *et al.*, 2016; 2018), diplonemids may be utilized for this purpose.

Therefore, the present study was performed to investigate the diplonemid community structure at two Japanese sites over two seasons and identify the possible cosmopolitan and endemic diplonemids by which the uniqueness of each community is characterized. Each community was characterized by an environmental DNA (eDNA) analysis, and environmental variables potentially influencing their community structures were examined. By comparing and characterizing communities with environmental variable data, we discussed similarities among them and the possible usage of diplonemids as indicators of environmental change.

## Materials and Methods

### Water collection, filtration, and an eDNA analysis

**Fig. 1.**
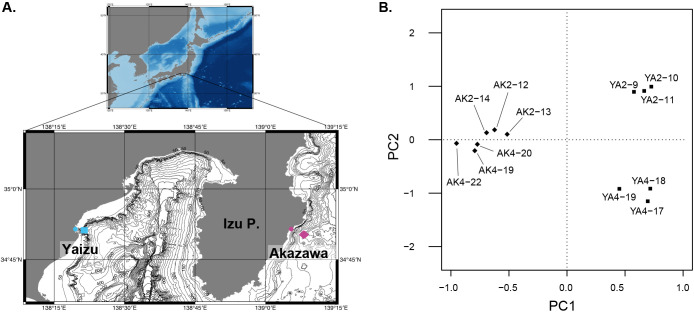
**A.** Map of Japan highlighting the location of deep-sea water pumping facilities (blue and purple dots) and the intake of deep-sea water (blue square and purple diamond) around the Izu Peninsula. Fig. 1A is modified from Fig. 1 in Yabuki *et al.* (2020). **B.** Principal component analysis of 12 replicates (three replicates from four samples).

Water samples were collected at the DHC deep-sea water pumping facility (Akazawa, Shizuoka, Japan) on September 25, 2019 and February 17, 2020, and at the Shizuoka prefectural deep-sea water pumping facility (Yaizu, Shizuoka, Japan) on September 26, 2019 and February 18, 2020 (Fig. 1A). Each water sample was collected independently three times (*n*=3), and the details of the collected samples are summarized in Table 1. Collected water was subsequently filtered on-site through a 0.45-μm Sterivex filter (Merck). After filtration, Sterivex tubes were filled with RNAlater (Life Technologies), kept on ice for 2–4 days during the expedition on-site, and then stored at –30°C in the lab until DNA extraction. Each Sterivex tube was gently opened and the filter was cut into pieces in accordance with the methodology described by Kawato *et al.* (2021). eDNA was extracted from the cut Sterivex filters using the DNeasy PowerSoil kit (Qiagen). Extracted DNA samples were diluted in 200‍ ‍μL of elution buffer. The sequences of diplonemids were amplified in accordance with the procedure described by Yabuki *et al.* (2020). To compare the sequences amplified from the same filtered water volume between the two sites, the DNA solution of Akazawa samples was diluted twice before amplification. Amplicon library construction and sequencing with a MiSeq Benchtop Sequencer (Illumina) were performed at Bioengineering Lab. Raw reads were subjected to QIIME2, and representative sequences were reconstructed (Bolyen *et al.*, 2019) with an initial quality check and the elimination of low-abundance sequences (*i.e.*, <0.1%). The representative sequences assigned to diplonemids as the blastn top hit were subjected to the analyses detailed below. The details of each sample are summarized in Table 1. Raw sequencing data were deposited in GenBank as DRA011389.

### Comparison of the diplonemid community among samples

Representative diplonemid sequences from all replicates were initially subjected to a principal component analysis (‘capscale’) using the Jaccard model, and the independent grouping of three replicates from each sample was tested by a permutational multivariate analysis of variance (PERMANOVA) (‘adonis’) using the Jaccard model with vegan 2.5–6 (Oksanen *et al.*, 2019) packaged-in R 4.0.3 (R Core Team, 2016) set in RStudio Version 1.3.1093 (RStudio Team, 2020). Sequences were then manually handled and categorized based on information regarding the existence/absence of each sample among the replicates. The distance matrix for the compositions of the sequences detected in each sample in total (collectively called ‘total series’) and those shared among all three replicates of each sample (collectively called ‘shared series’) were calculated by a clustering analysis (‘vegdist’) using the Jaccard model, and clustering was performed using the complete method. The sequences that were consistently detected in all samples were subjected to a phylogenetic analysis. These sequences were added to the existing alignment and aligned using MAFFT v7.032b with a default setting (Katoh *et al.*, 2002), and sites for the phylogenetic analysis were then selected using trimAl v1.4. rev22 with the strict option, *i.e.*, ‘-strict’ (Capella-Gutiérrez *et al.*, 2009). A maximum-likelihood tree was obtained from this dataset using RAxML v8.2.12 (Stamatakis, 2006) with the GTR+Γ+I model using bootstrap analyses (1,000 replicates). A Bayesian analysis was run using MrBayes v3.2.1 (Ronquist *et al.*, 2012) with the GTR+Γ+I model. Markov chain Monte Carlo (one cold and three heated) with default chain temperatures was run for 1×10^6^ generations with sampling trees at 100-generation intervals. The first 4×10^4^ generations were discarded as “burn-in” because it was confirmed that the scores of the sampled log-likelihood definitely plateaued before 4×10^4^ generations. All data including representative sequences and the analyzed dataset are available from the corresponding author upon request.

### Environmental variable data collection and statistical analyses

The water samples used to measure environmental variable data were collected at the same time as water collection for eDNA. The following variables were measured: temperature (°C), salinity (‰), electrical conductivity (mS cm^–1^), σT, the chl-a concentration (μg‍ ‍L^–1^), turbidity (FTU), and dissolved oxygen (DO; mg‍ ‍L^–1^) using a RINCO Profiler (JFE Advantech, Nishinomiya, Japan). The collected water depth referred to the information derived from each deep-sea water pumping facility (*i.e.*, 800‍ ‍m at Akazawa and 393‍ ‍m at Yaizu). Pearson’s correlation coefficient between each pair of variables was calculated using the ‘cor’ function in R 4.0.3 (R Core Team, 2016) set in RStudio Version 1.3.1093 (RStudio Team, 2020), and variables that correlated at more than |0.9| were omitted from statistical analyses. The results of the coefficient analysis are summarized in Table S1. Statistical analyses of environmental variable data were conducted using vegan 2.5–6 packaged-in R 4.0.3 set in RStudio Version 1.3.1093. The sequence composition data of the total and shared series were subjected to a distance-based redundancy analysis (RDA) (‘dbrda’) using the Jaccard model, and the significance of each variable on the community was tested using the ‘envfit’ function on the NMDS ordinating plot (‘metaMDS’).

## Results

**Fig. 2.**
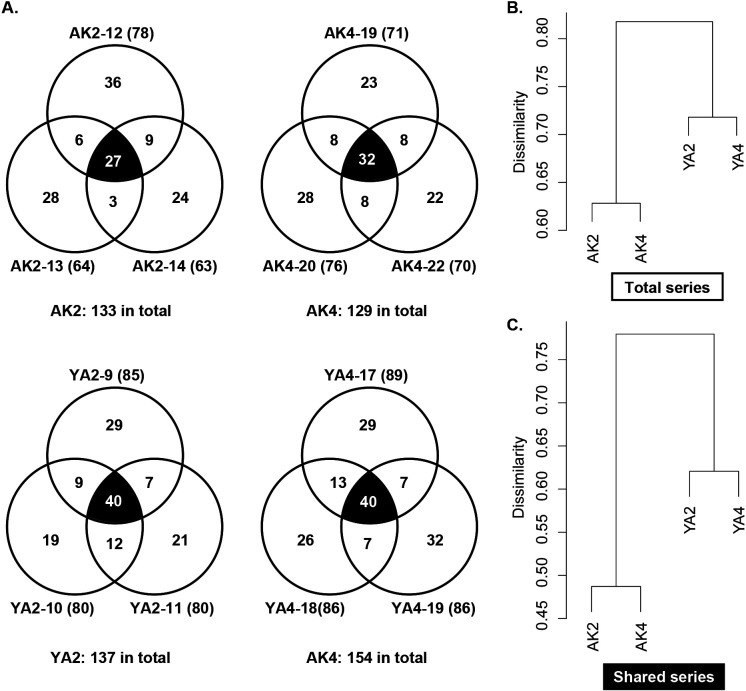
**A.** Venn diagrams showing the combination of three replicates in each sample. The numbers of representative sequences that are unique in each replicate or shared by two or three replicates are shown. The number of sequences shared by all three replicates of each sample is highlighted by white letters and a black background. The number of sequences retrieved in each replicate is shown in the bracket after the replicate name. **B** and **C.** Hierarchical clustering of four diplonemid community compositions in the total series (B) and shared series (C).

A total of 347 diplonemid representative sequences were retrieved from the sequencing analysis of 12 replicates. Principal component analyses showed four independent groups composed of three replicates from each sample (Fig. 1B), as revealed by PERMANOVA results (*P* value=0.002, *R^2^*=0.55426). The number of representative sequences in each sample (*i.e.*, the number in the total series) and that of the shared sequences among the three replicates (*i.e.*, the number in the shared series) are summarized in Fig. 2. A total of 133, 129, 137, and 154 sequences were retrieved from AK2, AK4, YA2, and YA4, respectively, and 27, 32, 40, and 40 sequences were commonly detected in all three replicates of each sample (Fig. 2A). The samples from the same locality clustered, and the dissimilarity between YA2 and YA4 was higher than that between AK2 and AK4 (Fig. 2B and C).

**Fig. 3.**
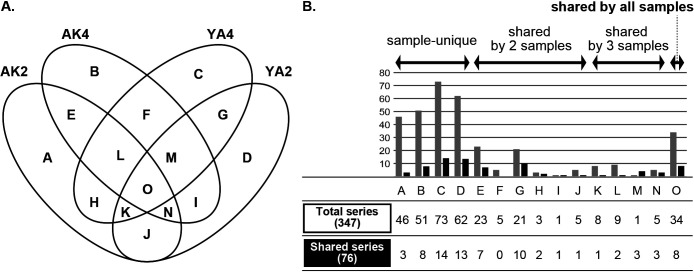
**A.** Venn diagrams showing the combination of four samples. The number of representative sequences unique to each sample or shared by two, three, or all four samples is shown. **B.** Bar graph and table showing the number of each category in Fig. 3A. The number of sequences in the total series and shared series is shown in gray and black, respectively, in the bar graph.

**Fig. 4.**
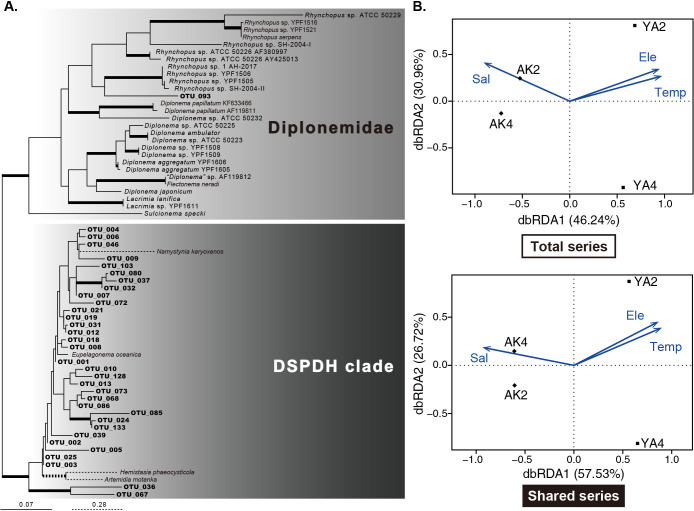
**A.** Phylogenetic tree of diplonemid 18S rRNA gene sequences. The sequences detected in category “O” in Fig. 3A are shown as OTU_#. The branches that are supported by bootstrap values ≥90% and Bayesian posterior probability ≥0.95 are shown by thick lines. **B.** and **C.** Redundancy analysis showing the co-relationship of four diplonemid community structures in the total series (B) and shared series (C) with the scores of environmental variables. Blue arrows indicate the environmental variables possibly influencing the diplonemid community structure. Sal, salinity; Ele, electrical conductivity; Temp, temperature. The first two and three axes explained 77.2 and 100% and 84.25 and 100% of the variability in the total series and shared series, respectively, in the fitted model.

In 347 total diplonemid representative sequences (and 76 sequences in shared series), 46 (3), 51 (8), 73 (14), and 62 (13) sequences were unique in each sample (corresponding to categories “A”, “B”, “C”, and “D” in Fig. 3A) and 34 (8) sequences were commonly detected in all samples (corresponding to “O”). The other 81 (30) sequences were shared with either two or three samples (corresponding to E, F, G, H, I, J, K, L, M, and N). The sequences shared between the same locality samples (*i.e.*, the summary of “E”, “L”, “N”, and “O” [=71 {20}] for AK2 and AK4, and the summary of “G”, “K”, “M”, and “O” [=64 {22}] for YA2 and YA4) were consistently more than those shared between the same season samples (*i.e.*, the summary of “J”, “K”, “N”, and “O” [=52 {13}] for AK2 and YA2 and “F”, “L”, “M”, and “O” [=49 {13}] for AK2 and YA4) (Fig. 3B). Of the 34 sequences that were classified into category “O” in the total series, 33 branched within the DSPDH clade, while only one (OTU_93) branched within the *Rhynchopus* clade in Diplonemidae (Fig. 4A). The tree topology and statistical support for each branching point were generally consistent with those reported by Yabuki *et al.* (2020), and major clades were well supported; however, the relationships among them were not resolved in detail (Fig. 4A).

Among the environmental variables that were measured in the present study (Table 2), salinity, temperature, electrical conductivity, the chl-a concentration, and turbidity were subjected to statistical analyses with Pearson’s correlation coefficient analysis (Table S1): σT and DO correlated with temperature and the chl-a concentration, respectively, and depth was omitted because the same locality samples shared the same value. The RDA showed that the distribution of four samples in both the total and shared series was fully explained by the first three axes in the fitted model (Fig. 4B and C). AK2 and AK4 were closely plotted and their separation from the other samples was strongly influenced by salinity (Fig. 4B and C). The sequence composition of YA2 was mainly influenced by electrical conductivity and temperature (Fig. 4B and C). However, their contribution to community structures was not significant (Table 3).

## Discussion

The present study showed that diplonemids were widely distributed in the targeted areas (63–89 OTUs from a single sample in this study); the number and diversity of the detected sequences were higher and more, respectively, than those reported by Lara *et al.*, 2009, who also analyzed deep-sea water samples (0–25 OTUs from a single sample). While recent studies in which diplonemid diversity was analyzed using NGS reported more sequences in total (*e.g.*, Flegontova *et al.*, 2016; 2020), this finding was obtained from an analysis of more samples and sequence diversity in the lineages in diplonemids was the same as that in the present study. At a much finer scale, the members comprising each diplonemid community were different from each other between the same locality as well as same season samples and even among the three replicates of a single sample (Fig. 1B, 2B, and 2C). While these differences were recognized in the present study, their sequence compositions were generally similar and the independent grouping of three replicates of each sample was statistically supported by PERMANOVA (Fig. 1B). Therefore, each replicate represented the diplonemid community in each sample, and the sequencing of a larger number of replicates contributed to the acquisition of more variable sequences. Our analyses showed that sequence compositions for the same season were dissimilar to each other rather than those at the same site (Fig. 2B and C); therefore, the diplonemid community may be conserved at the site during the season. However, some members emigrated from or immigrated to each sample, which contributed to the uniqueness of each community structure (Fig. 3A and B). It is important to clarify where and when these unique members originated because this information is key for understanding their distribution patterns. Although seasonal comparisons have not yet been conducted, the differences in diplonemid communities among many ocean samples were compared, and the possible influences of depth, DO, and other variables, such as salinity and latitude/longitude data, on diplonemid communities were also suggested (Flegontova *et al.*, 2020). Our RDA results also indicated that these variables influenced the diplonemid community structure around the Izu Peninsula; however, the significance of these influences was not statistically represented (Table 3). Further studies to compare more seasonal and site-rich samples by measuring more types of environmental variables are required to understand the principal element(s) regulating the community structure of diplonemids.

While the uniqueness of each community was shown, the diplonemids corresponding to category “O” in Fig. 4 may be widely distributed around the Izu Peninsula. Many members of this category phylogenetically belonged to the DSPDH clade, while *Rhynchopus* sp., a member of Diplonemidae, was also detected. The morphology and ultrastructure of *Rhynchopus* are distinguished from those of the other members of Diplonemea (Schnepf, 1994; von der Heyden *et al.*, 2004; Roy *et al.*, 2007; Tashyreva *et al.*, 2018); however, their ecological functions and roles are similar to those of other members. Although the physiological preferences of diplonemids, such as optimal growth temperature and speed, have not yet been compared, these differences may influence their distribution patterns, and members corresponding to category “O” may be more adaptive to the environments around the Izu Peninsula. Further studies are required for the discovery and culture establishment of unidentified members, which will contribute to our understanding of how diplonemids are widely distributed over the world’s oceans.

Although diplonemids have not yet been utilized as indicator organisms in the ocean environment, the possibility does exist. The diversity of diplonemids in each community is high and the distribution manner differs at the species/lineage level, which usefully contributes to the detection of differences and consistencies among communities. Furthermore, the distribution of diplonemids as a whole is cosmopolitan, which is also useful for comparing very diverse ocean samples with a focus on diplonemids. If the distribution of category “O” diplonemids is stable around the Izu Peninsula, their exchanges may only be caused by large environmental changes. Therefore, information on the presence/absence of possible adaptive members may help to monitor and detect environmental changes. Since diplonemids are not only a species(lineage)-rich, but also a high-biomass protist group in marine environments, it is possible to detect and compare the exchange of potentially cosmopolitan and conserved members to understand the indications of environmental changes. This viewpoint needs to be focused on and examined using more samples in future studies.

## Citation

Yabuki, A., Kawato, M., Nagano, Y., Tsuchida, S., Yoshida, T., and Fujiwara, Y. (2021) Structural Comparison of Diplonemid Communities around the Izu Peninsula, Japan. *Microbes Environ ***36**: ME21012.

https://doi.org/10.1264/jsme2.ME21012

## Supplementary Material

Supplementary Material

## Figures and Tables

**Table 1.** Summary of sequence and collected water information.

Sample ID	Replicate ID	Water collection date (time)	Filtration water volume (time)	Final DNA concentration (ng μL^–1^)	# of a set of pair reads	# of assembled reads	# of diplonemid reads (%)	# of diplonemid representative sequences
AK2	AK2-12	9/25/2019 (14:30, JST)	20 L (36‍ ‍min)	2.77*	56,872	23,235	23,057 (99.2%)	78
	AK2-13	9/25/2019 (14:31, JST)	20 L (35‍ ‍min)	2.75*	48,951	17,674	17,458 (98.8%)	64
	AK2-14	9/25/2019 (14:39, JST)	20 L (37‍ ‍min)	3.19*	49,241	18,684	18,607 (99.6%)	63
AK4	AK4-19	2/17/2020 (14:40, JST)	20 L (55‍ ‍min)	3.58*	48,206	18,336	18,254 (99.6%)	71
	AK4-20	2/17/2020 (14:40, JST)	20 L (55‍ ‍min)	3.75*	51,872	19,004	18,458 (97.1%)	76
	AK4-22	2/17/2020 (14:43, JST)	20 L (52‍ ‍min)	4.00*	50,364	19,226	18,584 (96.7%)	70
YA2	YA2-9	9/26/2019 (13:21, JST)	10 L (41‍ ‍min)	5.74	51,335	17,651	17,274 (97.9%)	85
	YA2-10	9/26/2019 (13:21, JST)	10 L (35‍ ‍min)	5.47	54,731	20,690	19,994 (96.6%)	80
	YA2-11	9/26/2019 (13:21, JST)	10 L (35‍ ‍min)	6.10	58,928	20,661	19,502 (94.4%)	80
YA4	YA4-17	2/18/2020 (12:10, JST)	10 L (38‍ ‍min)	7.02	51,383	18,290	17,748 (97.0%)	89
	YA4-18	2/18/2020 (12:10, JST)	10 L (42‍ ‍min)	7.21	56,458	22,421	21,887 (97.6%)	86
	YA4-19	2/18/2020 (12:10, JST)	10 L (41‍ ‍min)	7.46	54,784	21,891	21,552 (98.5%)	86

* DNA concentrations of AK2 and AK4 samples were measured after diluting twice.

**Table 2.** Summary of environmental data of collected deep-sea water.

Sample ID	Measurement date (and time)	Depth* (m)	Temperature (°C)	Salinity (‰)	Electrical conductivity (mS cm^–1^)	σ_T_	Chl-a concentration (μg L^–1^)	Turbidity (FTU)	DO (mg L^–1^)
AK2	9/25/2019 (13:01, JST)	800	7.191	34.424	34.936	26.939	4.23	79.34	4.721
AK4	2/17/2020 (14:19, JST)	800	7.200	34.37	34.894	26.894	0.41	77.82	1.982
YA2	9/26/2019 (14:12, JST)	393	10.645	34.259	37.966	26.261	2.64	89.26	4.715
YA4	2/18/2020 (12:13, JST)	393	9.005	34.184	36.401	26.474	1.03	133.4	3.391

* Depth data were not measured by RINKO-Profiler, but refer to information from each pumping facility.

**Table 3.** Summary of significance tests using the ‘envfit’ function

		Temperature	Salinity	Electrical conductivity	Chl-a	Turbidity
Total series	*P* value	0.1667	0.2083	0.1250	0.8333	0.2500
	*R^2^*	0.9749	0.9488	0.9759	0.3404	0.9913
Shared series	*P* value	0.1250	0.2917	0.1667	1.0000	0.2803
	*R^2^*	0.9936	0.3979	0.9882	0.0435	0.9905
