# Supplementary material for: Structural Comparison of Diplonemid Communities around the Izu Peninsula, Japan
Source: Microbes Environ. 2021 Jun 11;36(2):ME21012. doi: 10.1264/jsme2.ME21012 (PMC8209450; doi:10.1264/jsme2.ME21012)
Supplement: Supplementary file 1 — Supplementary Material [file 36_21012_s1.pdf]

Supplementary information

Table S1. Summary of Pearson's correlation coefficient analysis

	Temperature	Salinity	Electrical conductivity	σ_T	chl-a concentration	Turbidity	DO
Temperature	1.0	-0.8	0.8	-1.0	-0.2	0.6	-0.2
Salinity	-0.8	1.0	-0.6	0.8	0.4	-0.8	0.4
Electrical conductivity	0.8	-0.6	1.0	-0.8	0.4	0.8	0.4
σ_T	-1.0	0.8	-0.8	1.0	0.2	-0.6	0.2
chl-a concentration	-0.2	0.4	0.4	0.2	1.0	0.2	1.0
Turbidity	0.6	-0.8	0.8	-0.6	0.2	1.0	0.2
DO	-0.2	0.4	0.4	0.2	1.0	0.2	1.0

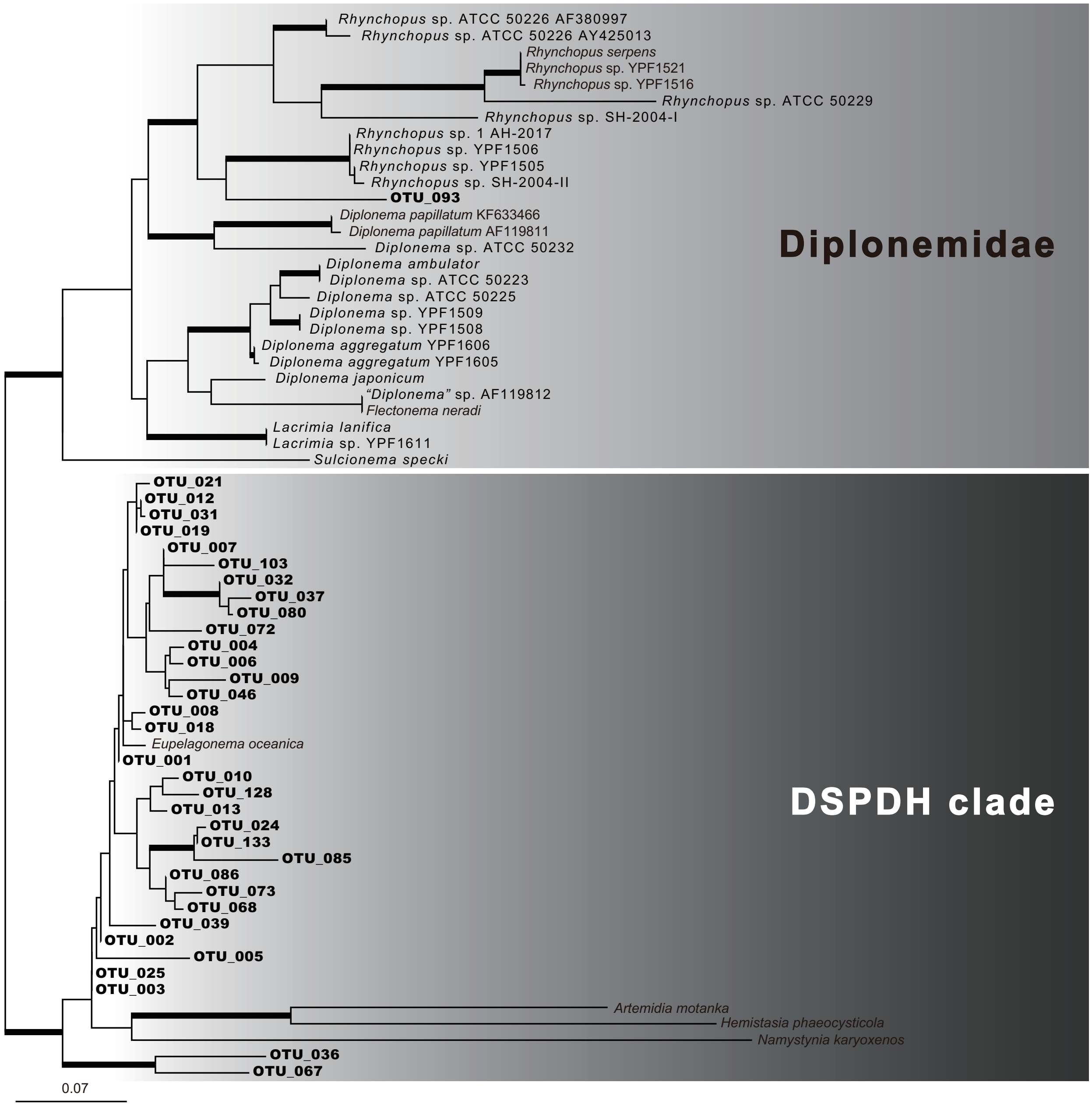

Fig. S1. Phylogenetic tree of diplonemid 18S rDNA sequences. The sequences detected in this study are shown as OTU_# and those belonging to category "O" in Fig. 3A are highlighted in white on a black background. The tree was obtained using IQ-TREE multicore version 1.6.12 (Nguyen et al. 2015) with TIM2e+G4 model, which was chosen by the best-fit model search potion ('-m TEST'). The branches that are supported by the standard nonparametric bootstrap analysis of 1,000 replicates ≥ 90%.

Reference:

Nguyen, L. T., Schmidt, H. A., Von Haeseler, A., and Minh, B. Q. (2015). IQ-TREE: a fast and effective stochastic algorithm for estimating maximum-likelihood phylogenies. *Mol Biol Evol* **32:** 268-274.
